# Genomic Prediction of Semen Traits in Boars Incorporating Biological Interactions

**DOI:** 10.3390/ijms252313155

**Published:** 2024-12-07

**Authors:** Yantong Chen, Fang Yang, Yanda Yang, Yulong Hu, Yang Meng, Yuebo Zhang, Maoliang Ran, Jun He, Yulong Yin, Ning Gao

**Affiliations:** 1 Key Laboratory of Livestock and Poultry Resources (Pig) Evaluation and Utilization, Ministry of Agriculture and Rural Affair, Yuelushan Laboratory, College of Animal Science and Technology, Hunan Agricultural University, Changsha 410128, China; chenyt0298@163.com (Y.C.); y621829@163.com (F.Y.); yangyanda_working@163.com (Y.Y.); huyulong1024864@163.com (Y.H.); 15188234010@163.com (Y.M.); ybzhangfd@hunau.edu.cn (Y.Z.); ranmaoliang0903@126.com (M.R.); hejun@hunau.edu.cn (J.H.); 2 Institute of Subtropical Agriculture, Chinese Academy of Sciences, Changsha 410125, China; 3 State Key Laboratory of Biocontrol, School of Life Sciences, Sun Yat-sen University, Guangzhou 510006, China

**Keywords:** boars, genomic prediction, semen traits, KEGG pathways, non-additive effects

## Abstract

In the context of modern pig farming, the central role of boars is underscored by large-scale centralized breeding and the widespread application of artificial insemination techniques. However, previous studies and breeding programs have focused mainly on product efficiency traits, such as growth rate, lean meat yield, and litter size, often neglecting boar semen traits. In this study, we estimated the genetic parameters and assessed the genomic prediction accuracy of boar semen traits with phenotypes evaluated from 274,332 ejections in a large population consisting of 2467 Duroc boars. Heritability of sperm morphological abnormality rate (ABN), fresh semen volume (VOL), sperm concentration (DEN), and motility (MOT) were estimated to be 0.43, 0.22, 0.23, and 0.16, respectively. GBLUP achieved a moderate predictive ability of semen traits, with a range of 0.32–0.50. Incorporating gene interactions indicated by the KEGG pathways (biBLUP) significantly improved predictive accuracy over the classical additive model (GBLUP) and epistatic model (RKHS). Moreover, biBLUP showed an improvement from 9.50% to 20.10% among the studied traits compared with GBLUP, with the greatest improvement (0.40 vs. 0.48) observed in sperm morphological abnormality rate. In conclusion, moderate to low heritability was estimated for the Duroc boar semen traits. Genomic prediction was able to achieve moderate accuracy, with a range from 0.32 to 0.56, for the studied traits. Considering gene interactions within KEGG pathways enhanced the predictive ability of boar semen traits.

## 1. Introduction

In recent years, with the widespread application of artificial insemination, the inheritance of superior boar genetics plays a crucial role in modern pork production systems. Traditional breeding programs have mainly focused on selecting traits such as growth rate, backfat thickness, and feed efficiency, neglecting the improvement of boar reproductive performance. Therefore, improving boar semen quality and enhancing boar reproductive performance are of urgent necessity. Previous studies reported that boar semen traits have shown moderate to low heritability, reflecting the potential of genetic improvement [1,2,3]. Genomic prediction (GP) [1], which utilizes genetic markers covering the whole genome to predict genetic merits, is able to achieve higher predictive accuracy than the traditional best linear unbiased prediction (BLUP) [4] and thus accelerate the genetic improvement progress of traits with low heritability.

With the development of high-throughput sequencing technology, the contribution of GP to the genetic improvement of economic traits in livestock and plants has become increasingly prominent, providing a basis for understanding the relationship between DNA sequence variations and economic traits [5,6,7]. GP is essential in facilitating trait selection, optimizing resource use, accelerating breeding progress, and promoting sustainable industry development [8]. Currently, statistical models used for GP include Genomic Best Linear Unbiased Prediction (GBLUP) [9], ridge regression best linear unbiased prediction (RR-BLUP) [10], Bayesian methods (BayesA, BayesB, etc.) [10,11], single-step GBLUP (ss-GBLUP) [7], and epistatic models such as reproducing kernel Hilbert space (RKHS) [12]. Depending on the genetic effects utilized, GP models can be categorized into additive effect models and non-additive effect models. Additive effect models assume that each gene’s impact on the phenotype is independent and cumulative, and they are widely used in livestock breeding. In contrast, non-additive effect models further consider gene–gene interactions, providing more accurate genetic parameter estimates and phenotype predictions. Ye, H. et al. H. et al. [13] introduced epistatic effects in genomic prediction using a haplotype-based model in Chinese yellow chickens, significantly improving prediction accuracy. Research in crops like maize [14] and wheat [15,16,17] also found that integrating non-additive effects could significantly enhance the accuracy of phenotype prediction. Moreover, it has been reported that models incorporating non-additive effects could achieve up to twice the predictive ability of additive effect models [18]. These findings demonstrate that models integrating non-additive effects have distinct advantages in phenotype prediction across different species, offering important theoretical guidance for further improving prediction accuracy. The rapid development and combined use of bioinformatics and molecular biology technologies have uncovered and validated the functions of numerous genes, providing resources for exploring gene interaction relationships. Previous studies suggest that estimating gene–gene epistatic effects based on prior gene function information and incorporating them into GP could significantly enhance prediction accuracy [19]. Aiming to incorporate gene interactions revealed by the KEGG (Kyoto Encyclopedia of Genes and Genomes) pathways into GP, Gao et al. [20] recently developed the biBLUP (biological interaction BLUP) model. This model uses KEGG pathways as carriers of biological interactions, integrating the interactive effects among genes within specific pathways into the model, thereby capturing these interactions in genomic prediction. This approach not only bridges the gap between statistics and biology but also enhances the predictive ability for complex traits. The model has improved the prediction accuracy by up to 40.36% and 16.29% for traits of yeast and rice, respectively. This indicates that integrating prior information regarding gene interactions in the GP model can enhance genomic prediction accuracy.

In this study, we estimated the genetic parameters of semen traits and investigated the genomic predictive ability in a large boar population with intensive phenotypic records and sequence level SNPs. Additionally, KEGG pathways were integrated into the prediction model to study the usefulness of pathways in modeling gene interactions and accelerating genomic prediction. This study intends to provide basic knowledge for constructing an early culling scheme for boars and further improve the efficiency of boar selection in large-scale pig breeding enterprises.

## 2. Results

### 2.1. Estimation of Genetic Parameters

The heritability of traits’ fresh semen volume (VOL), sperm concentration (DEN), motility (MOT), and abnormality rate (ABN) were estimated to be 0.221, 0.227, 0.164, and 0.432, respectively (Table 1). The parameter estimates indicate low to moderate heritability for the boar semen traits. Repeatability of traits VOL, DEN, MOT, and ABN were estimated to be 0.422, 0.363, 0.322, and 0.587, respectively.

**Figure 1:**
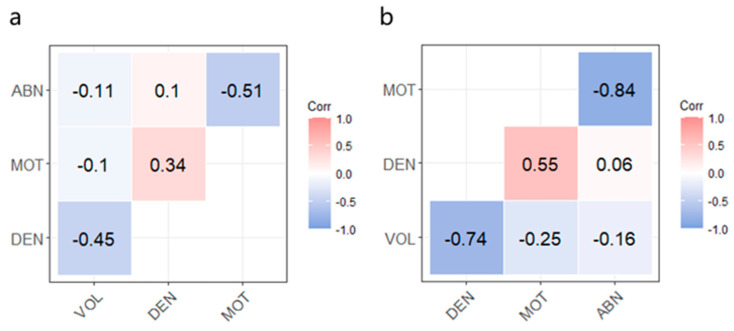
Genetic (**a**) and phenotypic correlations (**b**) of boar semen traits.

The estimates of genetic and phenotypic correlations for the four semen traits are presented in Figure 1. It was found that VOL is negatively correlated with DEN (−0.736), MOT (−0.248), and ABN (−0.20). This suggests that selecting boars with a larger fresh semen volume may result in reduced DEN, decreased MOT, and the reduced probability of ABN. However, from a biological perspective, there is typically a balance between VOL and DEN. In larger semen samples, the sperm count is often more dispersed, leading to a relatively lower DEN. DEN is positively correlated with MOT (0.552) and ABN (0.064). Higher DEN may be associated with healthier spermatogenic processes and maturation within the testes, with these sperm typically exhibiting stronger MOT and better movement capabilities. Additionally, higher DEN may reflect more optimal hormone levels (such as testosterone), which have a positive impact on sperm production and function. Environmental factors, such as appropriate temperature, nutritional support, and reduced external stress, also contribute to maintaining sperm quality, potentially enhancing the correlation between DEN and MOT. MOT and ABN are highly negatively correlated (−0.837), indicating that higher MOT is associated with a lower probability of ABN. This strong negative correlation provides valuable information for breeding. Sperm with higher motility generally have better quality and are less likely to exhibit morphological ABN. Therefore, in breeding programs, boars with higher MOT should be prioritized, as their sperm not only demonstrate better MOT but also have a lower incidence of morphological defects. This can be crucial for improving the reproductive performance of the herd. The estimates of phenotypic correlations among the four semen traits displayed similar trends as the genetic correlations (Figure 1).

### 2.2. Predictive Ability of the Classical Models

Overall, the genomic prediction of the semen traits demonstrated moderate predictive ability, with a range from 0.32 to 0.56 (Table 2), reflecting the potential of the implementation of genomic selection in the genetic improvement of these traits in elite boar breeding programs. For the studied boar semen traits, both GFBLUP and biBLUP showed an improvement in genomic prediction accuracy across all traits compared to GBLUP (Table 2). In this study, we found that the biBLUP method significantly outperformed the traditional GBLUP, RKHS, and GFBLUP methods in predicting boar semen traits. For the ABN trait, biBLUP was 20.10% more accurate than GBLUP. And it demonstrated superior predictive accuracy by 9.50% and 15.79% compared to RKHS and GFBLUP, respectively. This trend was similarly evident in the prediction of the VOL trait, where the biBLUP was 13.94% more accurate than GBLUP and performed 13.48% and 10.16% better than RKHS and GFBLUP. For the DEN trait, biBLUP showed the highest accuracy, outperforming GBLUP, RKHS, and GFBLUP by 13.63%, 12.39%, and 10.71%, respectively. In the prediction accuracy for the MOT trait, biBLUP similarly showcased its exceptional predictive power, leading by 15.63%, 10.12%, and 9.14% over GBLUP, RKHS and GFBLUP, respectively. These findings validate the potential of biBLUP in the field of genomic prediction.

### 2.3. Predictive Ability of the Top Pathways

In this study, we focused on investigating the utility of KEGG pathways in accelerating the prediction of semen traits. The top 10 KEGG pathways showed the highest predictive ability of fresh semen volume, as listed in Table 3. Comparatively, the biBLUP model, which accounts for interactions within the KEGG pathways, showed a better predictive accuracy than the GFBLUP model, with an increase ranging from 0.511 to 0.564. Overall, the biological functions encompassed by the top pathways include energy metabolism, cell cycle regulation, oxidative stress response, protein synthesis and degradation, tissue growth and regeneration, neurotransmission, metabolic regulation, immune response, cell proliferation and differentiation, and the development of blood and immune cells. These functions may indirectly or directly affect sperm vitality, maturation, survival, as well as the development and function of the testes, thereby exerting a significant impact on reproductive health and sperm quality. Notably, the beta-alanine metabolism pathway (ssc00410) showed the highest increase in predictive accuracy, representing a 10.37% increase compared with GFBLUP. Additionally, pathway ssc00410, which is involved in cellular energy metabolism and pH regulation, may indirectly influence sperm vitality and function.

For the trait of sperm concentration, the top pathways encompass a range of biological processes, including viral infections, protein digestion, glycosylation, autophagy, cardiovascular diseases, cytoskeletal dynamics, mRNA surveillance, platelet activation, drug addiction, and thermogenesis (Table 4). These biological functions may indirectly or directly influence sperm vitality, maturation, survival, as well as the development and function of the testes, thereby exerting a significant impact on reproductive health and sperm quality. The herpes simplex virus 1 infection pathway (ssc05168) showed the highest increase in predictive accuracy, representing a 11.18% increase compared to GFBLUP. This suggests a potential underlying connection between viral infection mechanisms and sperm function, possibly through the impact on the host’s immune response and cellular health.

For the trait of sperm MOT, the top pathways encompass biological processes including T cell receptor signaling, beta-alanine metabolism, retinol metabolism, Notch signaling, intestinal immune responses, morphine addiction, pancreatic cancer, and the degradation of branched-chain amino acids, as well as cytokine–cytokine receptor interactions (Table 5). These biological functions may indirectly or directly influence sperm motility, maturation, and survival, as well as the development and function of the testes, thereby exerting a significant impact on reproductive health and sperm quality. The identification of these pathways underscores the complexity of the genetic factors influencing sperm motility and highlights the importance of considering a broad spectrum of biological processes in genomic prediction models. Notably, the cellular senescence pathway (ssc04218) showed the highest increase in predictive accuracy, representing an 11.11% increase compared with GFBLUP. This suggests a potential underlying connection between cellular aging processes and sperm motility, possibly through impacts on the vitality and functional maintenance of sperm cells.

For the trait of sperm morphology abnormality, the top pathways encompass biological processes including VEGF signaling, long-term potentiation, metabolism of xenobiotics by cytochrome P450, insulin resistance, N-Glycan biosynthesis, FOXO signaling, basal cell carcinoma, antigen processing and presentation, and regulation of the actin cytoskeleton (Table 6). These biological functions may indirectly or directly influence sperm morphology, maturation, and survival, as well as the development and function of the testes, thereby exerting a significant impact on reproductive health and sperm quality. Notably, the pertussis pathway (ssc05133) showed the highest increase in predictive accuracy, representing a 16.91% increase compared to GFBLUP. This suggests a potential underlying connection between bacterial infection mechanisms, such as those involved in pertussis, and sperm morphology, possibly through the impact on the host’s immune response and cellular health.

## 3. Discussion

### 3.1. Estimation of Heritability for Semen Traits

Heritability is an important measure of the role of genetics in determining traits [21]. In this study, we estimated the heritability of semen traits in a large population of Duroc boars, providing valuable insights into the impact of genetics and the environment on traits. This is significant for further research into the mechanisms of trait formation and the prediction of phenotypes. Our results indicate that VOL and DEN possess moderate heritability, aligning with the results of pioneers in the field [1], suggesting that these traits can be effectively targeted in breeding programs to enhance the overall reproductive capacity of the population. For MOT, our results support the findings of previous researchers [22]. This discrepancy may be attributed to a broad range of variation in environmental factors [23] or potential estimation bias due to an insufficient sample size in previous studies [24]. The high heritability implies that selective breeding could significantly reduce the rate of ABN, thereby improving the overall reproductive quality of boars.

Compared to the study by Marques D et al. [22], the standard error of the estimated heritability in this study is lower, indicating the higher degree of precision and reliability of our estimates. Furthermore, we estimated the variance components using data from 2467 Duroc boars and 274,332 semen collections, a larger sample size than the study by Smital. J et al. [25], which involved 132 Duroc boars. The larger sample size reduces sampling error and improves the stability of the estimation, further enhancing the reliability of our results. This study indicates that incorporating gene interactions within KEGG pathways improved predictive accuracy, surpassing the traditional GBLUP and RKHS methods. Moreover, the biBLUP demonstrated a significant enhancement in predictive ability for the trait of ABN, further emphasizing the importance of enhancing the reliability of predictive models within the broader context of quantitative genetics.

### 3.2. Integration of Biological a Priori Information for Enhancing Genomic Prediction Accuracy

It is widely accepted in the quantitative genetics community that utilizing biological a priori information is a strategic approach to improve the accuracy of predictive models. Our results align with this consensus, suggesting that integrating biological a priori information into genomic prediction models is more likely to correctly identify biomarkers associated with the traits under study. Hashemi et al. [26] reported that by integrating some dynamical system properties, the predictive ability can be improved by reducing false-positive results. Additionally, the inclusion of gene annotations [27] or the genetic structure of traits [28] into genomic predictions was able to improve the predictive ability and reduce the prediction bias of traits in different species. Considering candidate gene identification, Zakharov et al. [29] highlighted the importance of biological information in clarifying variants that may significantly affect traits but are challenging to detect using conventional analyses, thereby enhancing the effectiveness of GWAS in uncovering associations between rare variants and complex traits. Our results further confirm the value of integrating diverse information sources in quantitative genetics research, especially when considering gene interactions. This indicates that carefully selecting and integrating biological a priori information can significantly improve the performance of genomic prediction models, which is of great importance for animal production.

### 3.3. Impact of KEGG Pathways on Gene Interaction Effects and Prediction Accuracy

In this study, we investigated the utility of KEGG pathways in capturing gene interaction effects and subsequently enhancing the predictive ability for semen traits in a large Duroc boar population. Compared with the classical additive effects model (GBLUP), the global interactive model (RKHS), and the additive model based on KEGG pathways (GFBLUP), the interaction effects model based on KEGG pathways (biBLUP) demonstrated varying degrees of improvement in predictive accuracy. Notably, the biBLUP model showed particularly prominent performance in the trait of sperm abnormality rate, with an improvement in predictive accuracy ranging from 9.14% to 20.10% over the classical models. These results indicated that the inclusion of non-additive effects significantly improved genomic prediction accuracy compared to models that only included additive effects. Our findings suggested a potential epistatic genetic background for the studied traits (especially for the trait of ABN) and highlighted the capability of KEGG pathways in capturing the underlying gene interactions that contribute to the variation in these traits.

Our study not only reveals the potential epistatic genetic background of these traits but also highlights the key role of KEGG pathways in deciphering these complex genetic interactions. Given the rapid advances in genomics and bioinformatics, we can now delve deeper into non-additive effects, which play a crucial role in determining multiple biological characteristics and have a far greater impact on economically important traits in animals than simple additive effects. The expression of many biological characteristics is influenced by the complex interactions between multiple genes [30]. Non-additive effects can explain a larger proportion of genetic variation compared to additive effects, and they play an important role in economically important traits in animals [31]. For instance, the genetic assessment and prediction of wood and growth traits were significantly improved by incorporating non-additive effects in the prediction model for tree growth traits in balsam fir [32]. By incorporating non-additive effects (such as dominance effects) into the prediction models for Holstein and Jersey cattle, the prediction accuracy of production traits was significantly improved compared to models that only considered additive effects [33]. These findings suggest that models integrating non-additive effects have significant advantages in phenotypic prediction. Moreover, genomic prediction models with non-additive effects can better capture the complex associations between genotypes and phenotypes, thereby enhancing prediction accuracy and stability, providing new ideas and methods for pig breeding practices [34].

### 3.4. Exploring the Role of Biological Pathways in Sperm Traits and Reproductive Efficiency

In this study, we utilized genomic prediction models, particularly the biBLUP model, to conduct an in-depth analysis of the genetic basis of semen traits in Duroc boars. By investigating predictive ability of the KEGG pathway model on the traits VOL, DEN, MOT, and ABN, we found the potential roles of multiple biological processes in spermatogenesis and sperm function. Firstly, we found that the beta-alanine metabolism pathway (ssc00410) showed an enhancement in predictive accuracy for semen volume, indicating that cellular energy metabolism and pH regulation may significantly influence sperm generation and vitality [35]. Notable, beta-alanine serves as an important intracellular buffering agent [36] and is crucial for maintaining the stability of the sperm cell environment. Additionally, beta-alanine metabolism was the most significant pathway in cauda epididymal lumen fluid [37]. Key enzymes within this pathway may participate in the energy metabolism of sperm, providing the necessary energy support for sperm motility and metabolic activities. Secondly, the significant improvement in predictive accuracy associated with the herpes simplex virus 1 infection pathway (ssc05168) for sperm concentration suggests that viral infections may indirectly affect sperm maturation and quantity by interfering with the host’s immune response and cellular health. A previous study had demonstrated that the asymptomatic seminal infection of HSV plays an important role in male infertility by adversely affecting sperm count [38]. HSV is not only detectable in semen but may also impair sperm quality, leading to male infertility or reduced fertility, and potentially increases the risk of miscarriage and adverse effects on the fetus when assisted reproductive technology (ART) is used [39]. Furthermore, the enhancement in predictive accuracy for sperm motility linked to the cellular senescence pathway (ssc04218) reveals that the process of cellular aging may impact sperm vitality and functional maintenance, subsequently affecting motility. Previous reports have indicated that cellular senescence, in synergy with the MAPK signaling pathway, further affects the normal synthesis of cholesterol and androgens, inhibits the normal synthesis of lactate and pyruvate, and ultimately affects spermatogenesis [40]. Cellular senescence can result in decreased energy metabolism and structural damage within sperm cells, thereby compromising their motility [41]. Lastly, the improvement in predictive accuracy for sperm morphological abnormalities was associated with the pertussis pathway (ssc05133), indicating that bacterial infections may influence sperm morphology and function by activating the host immune response. Bacterial infections can trigger the release of inflammatory mediators, which may exert toxic effects on sperm cells, leading to morphological abnormalities. Previous reports have indicated that treatment with pertussis toxin significantly reduces the ability of sperm to undergo the acrosome reaction [42].

Our results underscore the complex interplay of various biological processes in spermatogenesis and sperm function. These results not only enhance our understanding of the genetic basis of semen traits, but they also provide new perspectives for future reproductive health research and pig breeding practices. Through further functional studies and validations, these pathways may serve as potential targets for improving semen production and reproductive efficiency in boars. Future research could explore the regulatory mechanisms of specific genes and molecules within these pathways, as well as how they interact to influence semen traits. Additionally, genes and molecules within these pathways could act as biomarkers or therapeutic targets for developing strategies to enhance sperm quality and improve the success rate of artificial insemination.

## 4. Materials and Methods

### 4.1. Animals and Phenotypes

An expanded dataset from our previous GWAS study [43] with more boars and semen ejection records were involved in this study. Phenotypic records from the years between 2016 and 2020 of 274,332 ejections from 2467 Duroc boars (111 ejections each boar on average, with a range of 11–343) were analyzed. VOL, DEN, MOT and ABN were evaluated after ejaculation using the UltiMate^TM^ CASA system (Hamilton Thorne Inc., Beverly, MA, USA). The CASA system utilizes advanced digital image processing technologies to analyze and evaluate sperm motility and morphological characteristics. The system employs high-resolution microscopy and sophisticated image analysis software to perform the automated, in-depth analysis of sperm samples, including parameters such as sperm velocity, direction, morphology, and other biological factors. The CASA algorithms accurately identify and track the movement trajectories of individual sperm, enabling researchers to assess sperm quality in a highly efficient and objective manner. This approach enhances the precision and reliability of sperm quality evaluation. We removed any records where the VOL was less than 20 mL, any records with DEN higher than 20 × 10^9^/mL, any records where the MOT was less than 0.5, and any data where the ABN was more than 0.6. The outlier values were removed prior to final analysis. Overall, 7664 pigs were included in the pedigree. Boars with less than 10 ejection records were pre-pruned from the dataset. Descriptive statistics of the semen traits were presented in Table 7.

### 4.2. Genotyping, Sequencing, and Imputation

Among the 2467 boars with phenotypic records, 2261 of which include ear tissue or the fresh semen available were genotyped with the GeneSeek Genomic Profiler (GGP) 50K SNP array. SNP positions were matched to the Sus Scrofa 11.1 reference genome, leaving 48,829 autosome SNPs for further analysis. SNPs with minor allele frequency (MAF) ≤ 0.01 or call rate ≤ 90% were filtered out, and the remained markers were imputed through Beagle v5.1 [44] for further analysis.

Aiming at obtaining sequence level SNP data for further genetic study, we integrated a resequencing-imputation strategy in this study. Briefly, 95 highly representative individuals from the genotyped population were sequenced with depths of 10× coverage on average, and sequence-level SNPs were called via GATK [45]. SNP array data of the other boars were imputed to the sequence level with the sequenced individual serve as reference panel. Concerning the resequencing individual selection, we implemented the strategy introduced by Druet et al. [46]. Briefly, the kinship of the sequencing candidates was calculated based on genotype data, and the average kinship coefficients with other individuals were computed for each candidate. The first pig with the highest average kinship coefficient with the rest was chosen as the first sequencing subject. The remaining candidates were selected following the principle of “maximizing the representativity of the sequenced genomes” until an expected population genome representativity of 70% was achieved.

DNA was extracted from fresh semen (boars) or ear tissues (sows) of the selected 95 individuals and sequenced with 150 bp paired-end reads on the Illumina Novaseq6000 platform. GATK v4.0 [47] software was employed for SNP calling from the resequencing data. Initially, sequencing data were aligned to the pig reference genome (Sus scrofa 11.1) using the BWA software. SAMtools was used to convert sam files to binary bam files, and Picard’s MarkDuplicates was used to remove duplicate sequences. SNP detection and quality control were then conducted with GATK software, applying criteria such as Quality by Depth (QD) < 2.0, FisherStrand > 60.0, Mapping Quality (MAPQ) < 40.0, MQRankSum < −12.5, and ReadPosRankSum < −8.0. This resulted in 8.429 million high-quality SNPs.

GGP 50K chip data were imputed to sequence-level using Beagle v5.1 with the resequencing SNP panel as reference. The imputed sequence-level SNP data were further quality-controlled with the criteria MAF ≥ 0.01 and linkage disequilibrium $r^{2}<0.9$, resulting in 2084 boars and 262,766 SNPs for further analysis.

### 4.3. Genetic Parameter Estimation

The Asreml-R package was used to estimate breeding values for each trait using a repeatability model. Genetic parameters, including heritability ($h^{2}$), repeatability ($r^{2}$), and correlations among traits, were calculated. The four-trait repeatability model is given as follows:

(1) y=Xb+Age+Int+Zu+Ep+e,

where y is the matrix of observations, X is the incidence matrix for fixed effects, b is the vector of fixed effects of semen collection herd-year-season, and Age and Int denote the age of boar in month and collection interval, respectively. Z is the incidence matrix for polygenic effects; $u\sim N(0,A\sigma_{u}^{2})$ is the vector of estimated breeding values; A is the pedigree kinship matrix; E is the incidence matrix for permanent environmental effects; $p\sim N(0,I\sigma_{p}^{2})$ is the vector of permanent environmental effects; and $e\sim N(0,I\sigma_{e}^{2})$ represents the residuals, $\sigma_{u}^{2}$, $\sigma_{p}^{2}$, and $\sigma_{e}^{2}$, which are the variance components. De-regressed proofs (DRPs) were calculated following the formula introduced by Garrick [48], based on the estimated breeding values, for further predictive ability evaluation.

### 4.4. SNP-KEGG Pathway Mapping

The KEGGREST package [49] in R v4.2 was used to download the KEGG pathways for pigs (Sus scrofa 11.1) from the KEGG database. Genes were extended 5 kb upstream of the transcription start site and downstream of the transcription termination site to include potential regulatory elements. SNPs were mapped to genes and then to the KEGG pathways, resulting in SNP sets for all pathways [27]. KEGG pathways with fewer representative genes (each gene having at least one SNP located) than 10 were pruned from the analysis, resulting in 323 pathways for further study.

### 4.5. Genomic Prediction Models

This study intended to integrate KEGG pathways into the genomic prediction model and compare the predictive ability of the new model with the classical additive effect model (GBLUP), global epistatic model (RKHS), and additive effect model integrating single pathways (GFBLUP).

The GBLUP model is written as follows:

(2) y=μ+Zg+e,

where y is the vector of DRPs; μ is the overall mean, Z is the incidence matrix; $g\sim N(0,G\sigma_{g}^{2})$ is the vector of genomic estimated breeding values; $\sigma_{g}^{2}$ is the genetic variance; and $e\sim N(0,I\sigma_{e}^{2})$ represents the residuals, where $\sigma_{e}^{2}$ is the residual variance. The kinship matrix G is constructed according to VanRaden (2008) [9], $G=\frac{MM^{\prime }}{2\sum_{j=1}^{m}=1p_{j}\left(1-p_{j}\right)}$, where $p_{j}$ denotes the minor allele frequency (MAF) for the $j^{th}$ SNP; m denotes the total number of SNPs; and M denotes the MAF-adjusted genotype matrix with elements $\left\{0-2p_{j},1-2p_{j},2-2p_{j}\right\}$ representing genotypes AA, AB, and BB, respectively.

The model for RKHS [12] is the same as Equation (2) except that g is replaced with $i\sim N(0,K\sigma_{i}^{2})$, where i is the epistatic genetic values and $\sigma_{i}^{2}$ is the epistatic genetic variance. The Gaussian kernel (kinship) is calculated as $K\left(M_{i},M_{i^{\prime }}\right)=exp\{-\frac{||M_{i}-M_{i^{\prime }}|{|}^{2}}{h}\}$, where h represents the bandwidth parameter. In this study, we set the bandwidth parameter, following the form of our recent study [20], as $h=median\left(||M_{i}-M_{i^{\prime }}|{|}^{2}\right)/\{0.05,0.1,0.5,1,2,4\}$.

Concerning the KEGG pathway utilization, we compared two models with different manners for integrating the pathway information. For GFBLUP and biBLUP, additive or epistatic relationship matrices were constructed with SNPs mapped to each pathway, and subsequently, the additive or epistatic genetic values were calculated. The model incorporating KEGG pathways was defined as follows:

(3) y=μ+Zg+Zi+e,

where y represents is the vector of DRPs, μ represents the overall mean, and Z is the incidence matrix. Additionally, $g\sim N\left(0,G\sigma_{g}^{2}\right)$ is the same as defined in Equation (2), with $i\sim N(0,K_{p}\sigma_{i}^{2})$ representing the KEGG pathway (s) genetic values. For the GFBLUP [50,51,52] model, $K_{p}=G_{p}=\frac{M_{p}M_{p}^{\prime }}{2\sum_{j=1}^{mp}=1p_{j}\left(1-p_{j}\right)}$, $M_{p}$ denotes the MAF-adjusted genotype matrix of SNPs mapped to the $p^{th}$ pathway and mp denotes the number of SNPs mapped to the $p^{th}$ pathway. For biBLUP, $K_{p}$ is the Gaussian kernel calculated with the formula mentioned above using SNPs mapped to the $p^{th}$ pathway. If the target trait is genetically controlled by gene interaction effects and certain KEGG pathway can capture these interaction effects, then biBLUP is expected to outperform GFBLUP in predictive ability. The models mentioned above are summarized in Table 8.

### 4.6. Predictive Accuracy Assessment

The predictive ability of the genomic prediction models was evaluated through 20 repetitions of 5-fold random cross-validation. Predictive ability was defined as the Pearson’s correlation between the predicted total genetic values and the DRPs in the validation set.

## 5. Conclusions

In this study, we estimated genetic parameters and assessed the genomic predictive ability of semen traits in a large Duroc boar population. Moderate to high heritability and repeatability of ABN, VOL, DEN, and MOT were estimated. Moreover, genomic prediction achieved moderate accuracy of the studied semen traits, indicating the possibility of utilizing genomic selection in the genetic improvement of semen traits. Integrating KEGG pathways (biBLUP) showed a considerable improvement of predictive ability in the studied traits, especially for sperm morphological abnormality (ABN). To our knowledge, this study reported the genetic parameters and genomic predictive ability of semen traits based on the largest boar semen ejection records and number of boars in a single breeding population around the world so far.

## Figures and Tables

**Table 1:** Estimation of variance components and genetic parameters for semen traits.

Trait	$\sigma_{\alpha }^{2}\pm se$	$\sigma_{e}^{2}\pm se$	$\sigma_{pe}^{2}\pm se$	$h^{2}\pm se$	$r^{2}\pm se$
VOL	8.496 ± 1.017	22.269 ± 0.060	7.744 ± 0.680	0.221 ± 0.024	0.422 ± 0.009
DEN	1.217 ± 0.133	3.422 ± 0.009	0.732 ± 0.084	0.227 ± 0.022	0.363 ± 0.009
MOT	0.001 ± 0.000	0.003 ± 0.000	0.001 ± 0.000	0.164 ± 0.020	0.322 ± 0.008
ABN	0.003 ± 0.000	0.003 ± 0.000	0.001 ± 0.000	0.432 ± 0.030	0.587 ± 0.009

$\sigma_{\alpha }^{2}$: additive genetic variance; $\sigma_{pe}^{2}$: permanent environmental effect variance; $\sigma_{e}^{2}$: residual variance; $h^{2}$: heritability; $r^{2}$: repeatability; se: standard error; VOL: semen volume; DEN: sperm concentration; MOT: sperm motility; ABN: sperm morphological abnormality rate.

**Table 2:** Genomic predictive ability on semen traits in the Duroc breeding populations (Mean ± SE).

Traits	GBLUP	RKHS	GFBLUP	biBLUP
VOL	0.495 ± 0.002	0.497 ± 0.002	0.512 ± 0.020	0.564 ± 0.089
DEN	0.455 ± 0.002	0.460 ± 0.002	0.467 ± 0.011	0.517 ± 0.061
MOT	0.320 ± 0.002	0.336 ± 0.002	0.339 ± 0.013	0.370 ± 0.043
ABN	0.403 ± 0.003	0.442 ± 0.002	0.418 ± 0.016	0.484 ± 0.085

VOL: semen volume; DEN: sperm concentration; MOT: sperm motility; ABN: sperm morphological abnormality rate.

**Table 3:** Predictive ability of the top ten KEGG pathways for VOL (Mean ± SE).

Models	GFBLUP	biBLUP	Function
ssc00410	0.511 ± 0.021	0.564 ± 0.089	beta-alanine metabolism
ssc04068	0.512 ± 0.020	0.564 ± 0.089	FOXO signaling pathway
ssc00910	0.511 ± 0.021	0.563 ± 0.089	Nitrogen metabolism
ssc04390	0.511 ± 0.021	0.563 ± 0.089	Hippo signaling pathway
ssc04725	0.511 ± 0.021	0.563 ± 0.089	CHOLINERGIC SYNAPSE
ssc04950	0.511 ± 0.021	0.563 ± 0.089	Maturity onset diabetes of the young
ssc05150	0.511 ± 0.021	0.563 ± 0.089	Staphylococcus aureus infection
ssc05231	0.511 ± 0.021	0.563 ± 0.089	Choline metabolism in cancer
ssc04640	0.508 ± 0.014	0.542 ± 0.055	Hematopoietic cell lineage

The model denotes the identifiers for the KEGG pathways.

**Table 4:** Predictive ability of the top ten KEGG pathways for DEN (Mean ± SE).

Models	GFBLUP	biBLUP	Function
ssc05168	0.465 ± 0.011	0.517 ± 0.061	Herpes simplex virus 1 infection
ssc04974	0.466 ± 0.011	0.516 ± 0.061	Protein digestion and absorption
ssc00515	0.466 ± 0.011	0.516 ± 0.061	Mannose type O-glycan biosynthesis
ssc04136	0.466 ± 0.011	0.516 ± 0.061	Autophagy—other
ssc05410	0.465 ± 0.011	0.516 ± 0.061	Hypertrophic cardiomyopathy
ssc04814	0.466 ± 0.011	0.516 ± 0.061	Motor proteins
ssc03015	0.466 ± 0.011	0.516 ± 0.061	mRNA surveillance pathway
ssc04611	0.466 ± 0.011	0.516 ± 0.061	PLATELET ACTIVATION
ssc05032	0.467 ± 0.011	0.498 ± 0.041	Morphine addiction
ssc04714	0.465 ± 0.011	0.497 ± 0.041	Thermogenesis

The model denotes the identifiers for the KEGG pathways.

**Table 5:** Predictive ability of the top ten KEGG pathways for MOT (Mean ± SE).

Models	GFBLUP	biBLUP	Function
ssc04218	0.333 ± 0.013	0.370 ± 0.043	Cellular senescence
ssc04660	0.335 ± 0.013	0.365 ± 0.043	T cell receptor signaling pathway
ssc00410	0.334 ± 0.014	0.364 ± 0.044	beta-alanine metabolism
ssc00830	0.334 ± 0.014	0.364 ± 0.044	Retinol metabolism
ssc04330	0.334 ± 0.014	0.364 ± 0.044	Notch signaling pathway
ssc04672	0.334 ± 0.014	0.364 ± 0.044	Intestinal immune network for IgA production
ssc05032	0.334 ± 0.014	0.364 ± 0.044	Morphine addiction
ssc05212	0.334 ± 0.014	0.364 ± 0.044	Pancreatic cancer
ssc00280	0.334 ± 0.013	0.364 ± 0.043	Valine, leucine, and isoleucine degradation
ssc04060	0.333 ± 0.014	0.364 ± 0.043	Cytokine–cytokine receptor interaction

The model denotes the identifiers for the KEGG pathways.

**Table 6:** Predictive ability of the top ten KEGG pathways for ABN (Mean ± SE).

Models	GFBLUP	biBLUP	Function
ssc05133	0.414 ± 0.016	0.484 ± 0.085	Pertussis
ssc04370	0.418 ± 0.016	0.482 ± 0.085	VEGF signaling pathway
ssc04720	0.418 ± 0.016	0.480 ± 0.085	Long-term potentiation
ssc00980	0.416 ± 0.016	0.477 ± 0.085	Metabolism of xenobiotics by cytochrome P450
ssc04931	0.416 ± 0.016	0.474 ± 0.085	Insulin resistance
ssc00510	0.415 ± 0.016	0.471 ± 0.085	N-Glycan biosynthesis
ssc04068	0.414 ± 0.017	0.471 ± 0.085	FOXO signaling pathway
ssc05217	0.415 ± 0.016	0.467 ± 0.085	Basal cell carcinoma
ssc04612	0.414 ± 0.017	0.465 ± 0.058	Antigen processing and presentation
ssc04810	0.415 ± 0.017	0.461 ± 0.058	Regulation of actin cytoskeleton

The model denotes the identifiers for the KEGG pathways.

**Table 7:** Descriptive statistics for phenotypes.

Trait	Count	Minimum	Maximum	Mean	Standard Deviation	Coefficient of Variation (%)
VOL	2467	0.02	1.959	0.163	0.003	38.428
DEN	2467	0.01	19.97	5.063	2.303	45.485
MOT	2467	0.5	1	0.899	0.058	6.466
ABN	2467	0	0.6	0.0999	0.068	1.986

VOL: semen volume (L); DEN: sperm concentration (10^9^/mL); MOT: sperm motility; ABN: sperm morphological abnormality rate (%).

**Table 8:** Genomic prediction models in this study.

Model	Formula	Genetic Effects	Relationship Definition
GBLUP	y=μ+Zg+e	$g\sim N(0,G\sigma_{g}^{2})$	G: additive relationship matrix
RKHS	y=μ+Zi+e	$i\sim N(0,K\sigma_{i}^{2})$	K: Gaussian kernel
GFBLUP	y=μ+Zg+Zi+e	$g\sim N\left(0,G\sigma_{g}^{2}\right),i\sim N\left(0,G_{p}\sigma_{i}^{2}\right)$	$G_{p}$: pathway additive relationship
biBLUP	y=μ+Zg+Zi+e	$g\sim N(0,G\sigma_{g}^{2})$, $i\sim N\left(0,K_{p}\sigma_{i}^{2}\right)$	$K_{p}$: pathway Gaussian kernel

## Data Availability

The data presented in this study are available on request from the corresponding author for purely scientific (non-commercial) reasons.
